# Maternal mental health and trajectories of Preterm Behavioural Phenotype in infants born after a threatened preterm labour

**DOI:** 10.1192/j.eurpsy.2024.1691

**Published:** 2024-08-27

**Authors:** J. Andreu, J. Buesa, B. Almansa, C. Zapata, M. Lizarán, N. Gómez, F. Ghosn, A. Moreno, L. Campos, A. García-Blanco

**Affiliations:** ^1^Psychiatry, Hospital Universitario y Politécnico La Fe de Valencia; ^2^Psychology, La Fe Research Institute; ^3^Psychology, Hospital Universitario y Politécnico La Fe de Valencia, Valencia, Spain

## Abstract

**Introduction:**

Infants born preterm usually show a Preterm Behavioural Phenotype, which includes mixed symptomatology characterized by lack of attention, anxiety and social difficulties, with a 3-4 times greater risk of disorders in further childhood. Critically, this behavioural pattern is also observed in infants born after a threated preterm labour (TPL), regardless of the presence of prematurity. It is known that the course of this Preterm Behavioural Phenotype shows high variability. Nevertheless, the predictors of this Preterm Behavioural Phenotype prognosis remain unknown.

**Objectives:**

This study aimed to explore the predictors of change of Preterm Behavioural Phenotype symptomatology during preschool ages in order to improve prognosis.

**Methods:**

In this prospective cohort study, 117 mother–child pairs who experienced TPL were recruited. Preterm Behavioural Phenotype symptoms were assessed at age 2 and 6 using Child Behaviour Checklist. Gestational age at birth, maternal anxiety trait, maternal history of psychological traumas, prenatal and postnatal maternal depression, anxiety, and cortisol as well as parenting stress were included as predictors in a regression model.

**Results:**

Whereas increased internalizing problems were associated with a previous trauma history (p = .003), increased externalizing symptoms were linked to prenatal and postnatal maternal anxiety (p = .004 and p = .018, respectively).

**Conclusions:**

Identifying modifiable risk factors, such as the history of maternal traumas and anxiety at TPL diagnosis and postpartum is recommendable to enhance better prognosis of Preterm Behavioural Phenotype in the offspring.

**Disclosure of Interest:**

None Declared

